# FDG PET versus CT radiomics to predict outcome in malignant pleural mesothelioma patients

**DOI:** 10.1186/s13550-020-00669-3

**Published:** 2020-07-13

**Authors:** M. Pavic, M. Bogowicz, J. Kraft, D. Vuong, M. Mayinger, S. G. C. Kroeze, M. Friess, T. Frauenfelder, N. Andratschke, M. Huellner, W. Weder, M. Guckenberger, S. Tanadini-Lang, I. Opitz

**Affiliations:** 1 Department of Radiation Oncology, University Hospital Zurich and University Zurich, Rämistrasse 100, 8091 Zurich, Switzerland; 2 Department of Thoracic Surgery, University Hospital Zurich and University Zurich, Rämistrasse 100, 8091 Zurich, Switzerland; 3 Institute of Diagnostic and Interventional Radiology, University Hospital Zurich and University Zurich, Rämistrasse 100, 8091 Zurich, Switzerland; 4 Department of Nuclear Medicine, University Hospital Zurich and University Zurich, Rämistrasse 100, 8091 Zurich, Switzerland

**Keywords:** Radiomics, Machine learning, Artificial intelligence, Malignant pleural mesothelioma, Prognostic model, Clinical decision support system

## Abstract

**Background:**

Careful selection of malignant pleural mesothelioma (MPM) patients for curative treatment is of highest importance, as the multimodal treatment regimen is challenging for patients and harbors a high risk of substantial toxicity. Radiomics—a quantitative method for image analysis—has shown its prognostic ability in different tumor entities and could therefore play an important role in optimizing patient selection for radical cancer treatment. So far, radiomics as a prognostic tool in MPM was not investigated.

**Materials and methods:**

This study is based on 72 MPM patients treated with surgery in a curative intent at our institution between 2009 and 2017. Pre-treatment Fluorine-18 fluorodeoxyglucose (FDG) PET and CT scans were used for radiomics outcome modeling. After extraction of 1404 CT and 1410 FDG PET features from each image, a preselection by principal component analysis was performed to include only robust, non-redundant features for the cox regression to predict the progression-free survival (PFS) and the overall survival (OS). Results were validated on a separate cohort. Additionally, SUVmax and SUVmean, and volume were tested for their prognostic ability for PFS and OS.

**Results:**

For the PFS a concordance index (c-index) of 0.67 (95% CI 0.52–0.82) and 0.66 (95% CI 0.57–0.78) for the training cohort (*n* = 36) and internal validation cohort (*n* = 36), respectively, were obtained for the PET radiomics model. The PFS advantage of the low-risk group translated also into an OS advantage. On CT images, no radiomics model could be trained. SUV max and SUV mean were also not prognostic in terms of PFS and OS.

**Conclusion:**

We were able to build a successful FDG PET radiomics model for the prediction of PFS in MPM. Radiomics could serve as a tool to aid clinical decision support systems for treatment of MPM in future.

## Background

Malignant pleural mesothelioma (MPM) is an aggressive thoracic malignancy with a dismal prognosis. The tumor originates from cells of the visceral or parietal pleural and is linked to asbestos exposure with a median latency of 44.6 years [1]. Due to the latency between exposure and onset of mesothelioma and the ongoing use of asbestos in parts of the world, the incidence is expected to rise continuously in the next years, necessitating improvements in management of these patients. Life expectancy is still poor today, with a median overall survival of approximately 12 months [2]. Multimodal treatment strategy is associated with a prolonged median survival of up to 29 months, but also harbors the risk of increased toxicity [3, 4]. Adjuvant radiation therapy after chemotherapy and radical surgery was investigated in a multicenter phase II trial and did not show a benefit for locoregional relapse-free survival and thus cannot be considered as a standard adjuvant treatment for MPM patients [5]. To date, most centers offer multimodal treatment consisting of (neo-)adjuvant chemotherapy in conjunction with maximal surgical cytoreduction [6]. However, only a minor subset of all newly diagnosed patients is considered to be eligible for such radical surgery. The vast majority of patients receive palliative systemic therapy. Careful selection of appropriate candidates for a curatively intended and potentially toxic multimodal treatment is of highest importance, asking for prognostic factors and scores. There are some known clinical prognostic factors, such as the performance status and histology among others [7], that are incorporated into the EORTC–prognostic score. Based on this, a discrimination of pleural mesothelioma patients into a good- and a poor-prognosis group is possible [8].

Recently, morphological features derived from medical images were discovered as additional important prognostic factors. Tumor volumetry and maximal pleural thickness on axial CT slices were prognostic in terms of median survival [9, 10]. The International Association for the Study of Lung Cancer (IASLC)/International Mesothelioma Interest Group (IMIG) database reported a correlation between the maximal pleural thickness on axial CT slices and T stage (according TNM 7th edition), overall stage, nodal stage, and survival [11].

Radiomics is an advanced computational method to describe tumors in a more comprehensive way than simple measurements. Shape, intensity, and texture of a tumor are quantified on medical images through mathematical analysis, resulting in hundreds of extracted features [12]. By applying mathematically defined filters, even more information can be extracted from images by, e.g., enhancing high and low frequency components of the images, such as edges and reduced noise. Radiomic signatures were shown to be prognostic for survival and local tumor control in multiple tumor entities [13]. However, MPM has not been investigated yet using this approach. The aim of our study was to analyze the prognostic ability of CT-based and Fluorine-18 fluorodeoxyglucose (FDG) PET-based radiomics models for the prediction of progression-free-survival (PFS) and overall survival (OS) in MPM patients undergoing a curative treatment approach.

## Methods

### Studied population

In total, 72 MPM patients were studied retrospectively out of 123 patients referred for treatment to the University Hospital Zurich between 2009 and 2017. Clinical parameters and initial pre-therapeutic staging by FDG PET/CT were available. Confirmation of diagnosis by histological examination of biopsy specimens was available in all subjects. All patients underwent curative treatment consisting of at least aggressive surgery. Induction chemotherapy with platinum and pemetrexed, administered for 3 to 4 cycles, was performed in 60 out of 72 patients. Curative surgery was performed either by extrapleural pneumonectomy (EPP) or pleurectomy/decortication (P/D). The training and the validation cohort consisted of 36 patients each (split by date of treatment). Median follow-up was 51.9 months (22.4–70.5 months) and 24.1 months (13.5–39.8 months), overall survival (OS) was 21.5 months (2.6–74.8 months) and 23.7 months (5.9–39.8 months) and PFS was 11.3 (range 2.6–51.9 months) and 11.7 (5.0–39.8 months) for training and validation cohort, respectively. Detailed patients’ characteristics are provided in Table 1.

**Table 1:** Patient characteristics

	Training cohort (*n* = 36)	Validation cohort (*n* = 36)
Age (range)	64 (40–67)	66 (49–76)
Gender (%) - Male - Female	34 (94) 2 (6)	30 (83) 6 (17)
Histology (%) - Epithelioid - Biphasic - Sarcomatoid	31 (86) 4 (11) 1 (3)	30 (83) 5 (14) 1 (3)
Surgery (%) - P/D - EPP	23 (64) 13 (36)	30 (83) 6 (17)
Induction chemotherapy (%)	25 (69)	35 (97)

*P/D* pleurectomy/decortication, *EPP* extrapleural pneumonectomy

### Image acquisition and definition of volumes

For all patients, pretreatment FDG PET and native CT scans were available. Blood glucose level was measured prior to FDG PET/CT. All PET scans were corrected for decay, attenuation, scatter, dead time, and random. Details on scanning parameters are provided in Table 2. To reduce variability in imaging acquisition between patients, non-contrast-enhanced CT scans were used as some patients did not receive contrast due to various reasons. Manual delineation of the primary tumors was performed by four radiation oncologists (with more than 3 years of experience) on co-registered CT and FDG PET images according to a study-specific protocol: all FDG PET-positive masses were included as well as FDG PET-negative but highly suspicious pleural thickenings, lung nodules, infiltrated pericardium and mediastinal extension on CT imaging. Pleural effusion and atelectasis were excluded. Contouring was performed either in Eclipse (Varian Medical Systems VR, Palo Alto, CA) or MIM Vista (Version 6.7.9, MIM Software Inc. VR, Cleveland, OH).

**Table 2:** Scanning parameters

Scanning characteristics/parameters	Training cohort (= 36)	Validation cohort (= 36)
CT scanners	Siemens Biograph40 (*n* = 9) GE Discovery STE (*n* = 7) GE Discovery 690 (*n* = 15) GE Discovery VCT (*n* = 5)	Siemens Biograph40 (*n* = 16) Siemens Biograph128 (*n* = 4) GE Discovery STE (*n* = 3) GE Discovery 690 (*n* = 3) GE Discovery VCT (*n* = 7) GE Discovery 600 (*n* = 1) GE Discovery MI (*n* = 2)
- Slice thickness (mm)	2.5–4	1.25–3.27
- In-plane resolution (mm)	0.98–1.52	0.98–1.52
- kV	100; 120; 140	100; 120; 140
- mAs	62–402	23–136
- Reconstruction kernel	Soft kernel (*n* = 33) Sharp kernel (*n* = 3)	Soft kernel (*n* = 20) Sharp kernel (*n* = 16)
PET scanners	Siemens Biograph40 (*n* = 9) GE Discovery STE (*n* = 7) GE Discovery 690 (*n* = 15) GE Discovery VCT (*n* = 5)	Siemens Biograph40 (*n* = 15) Siemens Biograph128 (*n* = 4) GE Discovery STE (*n* = 3) GE Discovery 690 (*n* = 4) GE Discovery VCT (*n* = 7) GE Discovery 600 (*n* = 1) GE Discovery MI (*n* = 2)
- Slice thickness (mm)	2.5–4	2–3.27
- In-plane resolution (mm)	2.73–5.47	2.73–5.47
- Administered FDG activity (MBq), median (range)	337.5 (188–417)	316.3 (204.1–408)
- Delay between administration of FDG and scanning (min)	46.1–85.6	49.8–91.1
- Reconstruction algorithm	3D OSEM (*n* = 18) 3D OSEM with PSF (*n* = 18)	3D OSEM (*n* = 24) 3D OSEM with PSF (*n* = 12)

*kV* kilovolt, *mAs* milliampere-second, *MBq* Mega Becquerel, *OSEM* ordered subset expectation maximization., *PSF* point spread function modeling

### Image pre-processing and radiomics analysis

The in-house developed radiomics software Z-rad written in Python programing language (version 2.7.6) was used to analyze the 3D images by extracting shape, intensity, texture, and wavelet features. This software package was benchmarked in the Image Biomarker Standardization Initiative [14]. For intensity, texture, and wavelet analysis, images were resized to cubic voxels of 3.3 mm in CT and 5.5 mm in PET using linear interpolation. These voxel sizes correspond to the most common image resolution in CT (sagittal) or PET (axial). Additionally, Hounsfield units (HU) range − 300 to 200 in CT was applied to limit the analysis to tumor tissue only. These adapted CT contours were then transferred to the PET images. To quantify the texture and wavelet, images were discretized to equally spaced bins of 5 HU in CT and 0.25 SUV in FDG PET.

In total, 1404 features from CT images and 1410 features from FDG PET images were extracted, according to Pavic et al [15]. All details on definitions and description of analyzed features are provided in that paper and online on the website giving a detailed overview on the radiomics software including code and definition of features [16]. Six additional features for the FDG PET imaging described volumes exhibiting metabolism above certain threshold of the maximum SUV (metabolic tumor volume 20%, 30%, 40%, 50%, 60%, 70%). To account for differences in contouring between the different observers, only stable features irrespective of differences in tumor contouring were considered for further analysis. For CT images, this analysis was done on 11 cases out of the entire cohort in a previous work and a detailed list of all extracted as well all stable CT features used for modeling is provided in supplementary material of the above-mentioned inter-observer delineation variability study [15]. For FDG PET images, the analysis on stable features was done prior to feature extraction on the same 11 MPM cases and following the same procedure as for the CT study. In brief, for each region of interest, the radiomics analysis was performed and consistency of the three respective results was tested using the intraclass correlation coefficient (ICC), whereupon an ICC > 0.8 was accepted as a value to indicate robustness [17]. The description of procedures is detailed in a publication on CT scans by Pavic et al. [15].

### Statistical analysis

Statistical analysis was performed in R (version 3.3.2). OS and PFS were determined from the date of initial diagnosis. First, features with more than 20% missing values or low variability were excluded from the analysis. The remaining features were grouped using principal component analysis, and the Horn method was used for definition of the optimal number of retained components [18]. Univariable Cox regression analysis was applied to determine prognostic value of correlated features. Per principal component related group, the feature with the highest Concordance Index (c-index), and corresponding false discovery rate < 0.25 in the univariable Cox regression was selected. The prognostic non-redundant features (one feature per principal component group) were entered in the multivariate Cox regression analysis with backward selection of variables using Akaike information criterion. For the split into risk groups, we used 80th percentile of the predictions in the training cohort, yielding a threshold of 0.35. We have chosen the split percentile based on the most significant result in the training cohort. The model was validated in the separate cohort of 36 patients. The risk group stratification was studied with the G-rho test. A *p* value below 0.05 was considered significant.

**Fig. 1:**
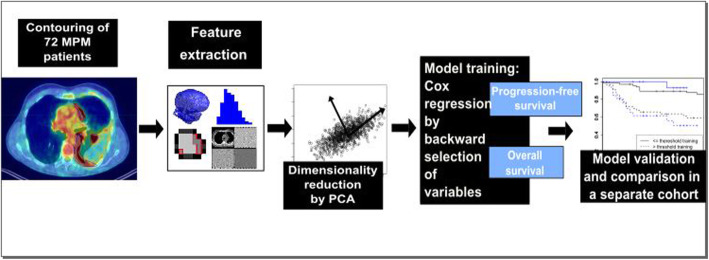
Overview of radiomics workflow

In order to test the added value of a radiomics analysis over routinely gathered information by PET-CT, that was already shown to have prognostic value, we calculated the prognostic power of standard uptake value (SUV) max and SUV mean and of volume for PFS and OS [9, 19] (Fig. 1).

## Results

### Robustness of FDG PET-radiomics

In the cohort of 11 MPM patients, on which the stability of CT radiomics results according to the inter-observer delineation variability was tested previously, 780 out of 1410 FDG PET features (55.3%) were stable against variability in tumor segmentation. The stable features included 1 shape (5.6% shape features), 20 intensity (87% intensity features), 82 texture (59.9% texture features), and 677 wavelet features (55% wavelet features). Additional file 1: Tables 1, 2, 3, and 4 provide the full list of stable features.

### CT radiomics model

For the PFS, dimensionality reduction by PCA derived five groups of correlated features in the training cohort. Only one group contained features with a good discriminative power. The final model consisted of one radiomic feature: “LHH GLRLM long run high grey level emphasis” (a wavelet feature).

For the OS, dimensionality reduction by PCA derived five groups of correlated features, that all contained features with a good discriminative power. After backward selection, the final model consisted of three radiomic features: “GLSZM grey level non-uniformity” (a texture feature),”HLH GLCM homogeneity” (a wavelet feature), and “LHH intensity range” (a wavelet feature). However, for both PFS and OS, the model could not be successfully validated (see Table 3). Thus, no CT radiomics model with a good prognostic ability could be generated.

**Table 3:** Overview of modeling results

Performance of model	PFS	OS
FDG PET-model, c-index (95% CI) Training Validation	0.67 (0.52–0.82) 0.66 ( 0.57–0.78)	0.72 (0.62–0.84) 0.47 (0.36–0.62)
CT-model, c-index (95% CI) Training Validation	0.66 (0.56–0.76) 0.54 (0.44–0.67)	0.71 (0.59–0.80) 0.59 (0.47–0.74)
SUVmax, c-index (95% CI) Training Validation	0.55 (0.44–0.60) –	0.54 (0.42–0.68) –
SUVmean, c-index (95% CI) Training Validation	0.52 (0.40–0.66) –	0.52 (0.40–0.66) –
Volume, c-index (95% CI) Training Validation	0.60 (0.49–0.73) 0.57 (0.48–0.67)	0.62 (0.52–0.72) 0.63 (0.50–0.75)

*PFS* progression-free-survival, *OS* overall survival

### FDG PET radiomics model

**Fig. 2:**
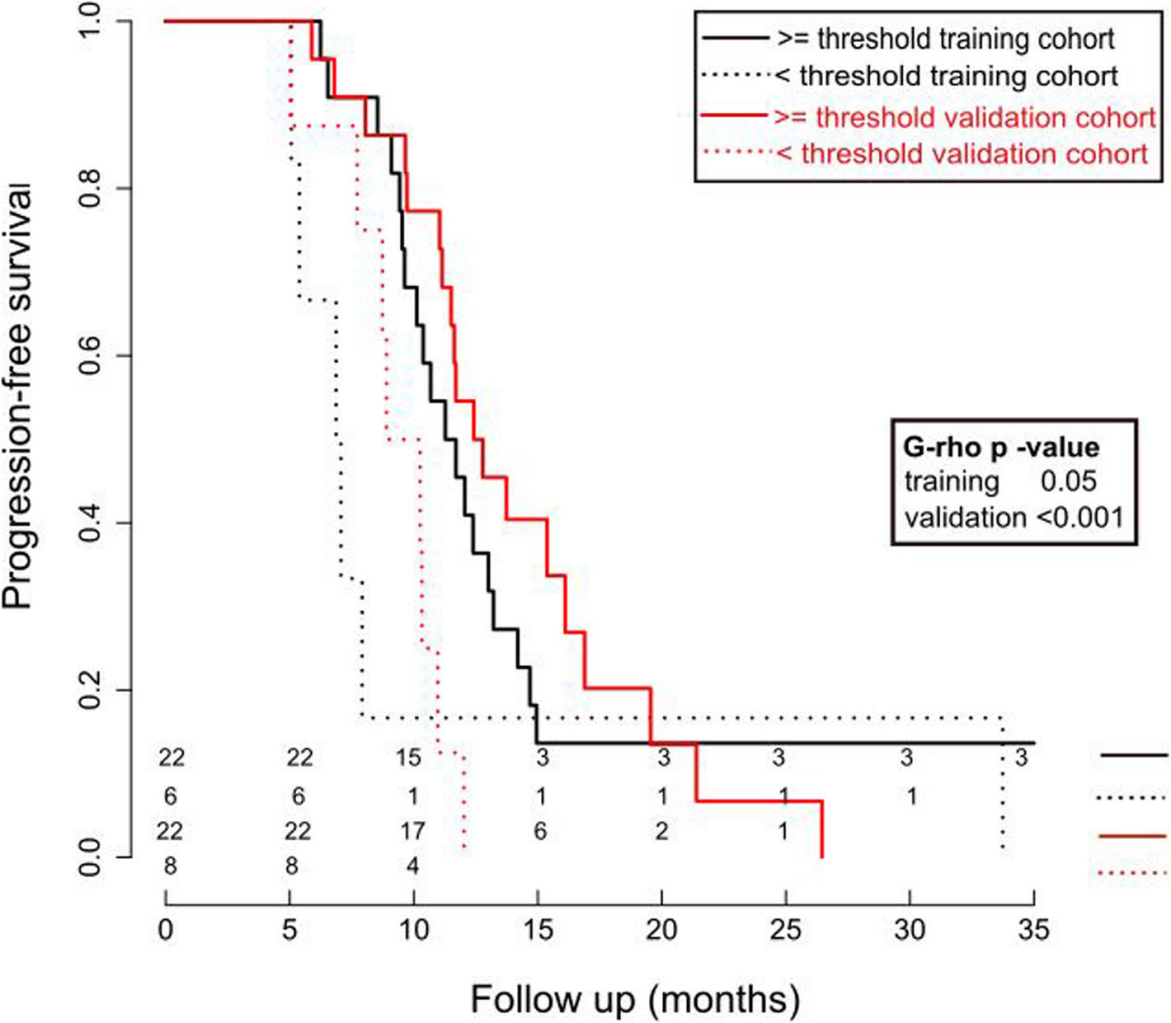
Cox regression results for FDG PET radiomics model for PFS

For the PFS, dimensionality reduction by PCA resulted in three groups of correlated FDG PET features in the training cohort. All three groups contained features with a good discriminative power. After backward selection, the final model consisted of three radiomic features: “HLH intensity range,” “HLH GLSZM high grey level zone emphasis,” and “HLH GLCM maximal correlation coefficient.” All three features represent wavelet features. The model performance was first estimated in 5-fold cross validation in the training cohort with a mean c-index of 0.67 (95% CI 0.52–0.82). The performance of the model for the validation cohort showed a good prognostic power with a c-index of 0.66 (95% CI 0.57–0.78). The model splits the patients into groups with significantly different PFS in the training (11 vs. 7 months, *p* 0.05) as well as in the validation (12.5 vs. 9 months, *p* < 0.001) cohort (see Fig. 2). Our PFS PET radiomics prognostic model showed also good discrimination for OS with c-index = 0.66 (95% CI 0.52–0.80). Details for the Cox model are provided in Additional file 3 in supplementary material.

For the OS, dimensionality reduction by PCA resulted in three groups of correlated FDG PET features in the training cohort. All three groups contained features with a good discriminative power. After backward selection, the final model consisted of two radiomic features: “LHH GLSZM small zone high grey level emphasis” and “LLL GLCM information measures of correlation 2”—both representing wavelet features. The model performance first estimated in 5-fold cross validation in the training cohort showed a mean c-index of 0.72 (95% CI 0.62–0.84). The performance of the model for the validation cohort showed a poor prognostic power with a c-index of 0.47 (95% CI 0.36–0.62).

### SUV and volume results

For the PFS and OS, the performance of SUVmax was c-index = 0.55 (0.44–0.60) and c-index = 0.54 (0.42–0.68), respectively. Performance of SUVmean for PFS was c-index = 0.52 (0.40–0.66) and for OS c-index = 0.48 (0.39–0.61). Thus, on the training cohort, no prognostic model could have been built based on SUVmax and SUVmean.

The volume-based model showed no prognostic performance for PFS with a c-index of 0.60 (0.49–0.73) and a c-index of 0.57 (0.48–0.67) for the training and validation cohort, respectively. Yet, for the OS, the model was prognostic with a c-index of 0.62 (0.52–0.72) and a c-index of 0.63 (0.50–0.75) for the training and validation cohort, respectively (see Additional file 2: Figure 1). See Table 3 for an overview of results.

## Discussion

We were able to train a radiomics model on pre-treatment FDG PET images being predictive for the PFS. The significant but in terms of absolute numbers moderate PFS advantage translated also into an OS difference when applying the radiomics model developed on the PFS to test for the OS differentiation. On the contrary, a simple model based on SUVmax or SUVmean had no prognostic power for PFS nor OS. Volume was prognostic for the OS but showed lower c-index than the FDG PET radiomics-based PFS model.

The ability of radiomics on CT and MRI images to support the diagnostic process of differentiation between benign and malignant pleural lesions has recently been proposed with an AUC of 0.92 for the CT model and AUC of 0.87 for the MR model [20]. However, to the best of our knowledge, no radiomics model for outcome prediction in pleural mesothelioma patients has been reported yet. The Multimodality Prognostic Score (MMPS; range 0–4) was generated to identify patients which may benefit most from an aggressive multimodal treatment [10]. Using the following prognostic factors, the MMPS could distinguish patients with different prognosis in terms of OS: tumor volume pre-chemotherapy (pre-CTX) > 500 ml, CRP pre-CTX > 30 mg/l, non-epithelioid histology in pre-CTX biopsy, and progressive disease according to modified RECIST criteria [21]. Patients with a MMPS of ≥ 3 did experience a significantly shorter OS. Our cohort consisted of patients all being eligible for aggressive surgery, and we had no patient with an MMPS of ≥ 3 included—therefore, a comparison of the radiomics model with the MMPS score is not possible. The MMPS allows selection of patients for referral to surgery after induction chemotherapy. Our radiomics model was developed on medical images acquired before any treatment was initiated and could therefore in future contribute to a prediction model and decision-support system for individualized treatment strategy before initiation of induction chemotherapy. A notable advantage of prediction models based on images is that these medical images are routinely acquired for diagnostic purposes—the ASCO guidelines recommend an FDG PET/CT as staging method for all MPM patients considered candidates for definitive surgical resection [6]—and therefore are available for all patients without the need for an additional procedure.

The final FDG PET radiomics model for prediction of PFS comprised three wavelet features, “HLH intensity range,” “HLH GLSZM high grey level zone emphasis,” and “HLH GLCM maximal correlation coefficient.” All the features in the final model were extracted from the HLH wavelet map [22]. The HLH wavelet filtering emphasizes the SUV heterogeneity in 2 out of 3 dimensions. The maximal correlation coefficient is a correlation measure and high grey level zone emphasis takes high values in the images with larger patches of high intensity. For both of those features, higher values were associated with worse prognosis. The intensity range corresponds to the range of wavelet coefficients in the ROI. In contrary to other features, the lower intensity range was associated with worse prognosis.

A strength of our study is the implementation of radiomics for MPM. This tumor is characterized by a very heterogeneous shape and diffuse growth along the pleura and frequent infiltration of thoracic structures. Therefore, this tumor is difficult for contouring. A prerequisite for implementation of a radiomics model as a decision-making tool into clinical routine is robustness of every individual step in the process—one important step in the radiomics workflow is segmentation of the region of interest. Inter-observer variability in contouring of the tumor was investigated for several sites and can be substantial [23, 24]. To account for this uncertainty, we used only features in our model, which are robust irrespective of variations in contouring [15]. In total, 505 features were used for CT and 780 for FDG PET radiomics in the current analysis. Thus, a higher proportion of FDG PET features was stable compared to CT features. The potential reason for the higher percentage of stable features lies within the imaging modality itself: high-FDG uptake in PET images is quite apparent and contouring variability is expected to occur mainly in rim regions where low-FDG uptake or blurring is present. This rim region represents only a minor sub-volume compared to the whole FDG uptake area and therefore, uncertainties in this region do not lead to a high variability of radiomics results. Another possible factor is the bigger voxel size of 5.5 mm in FDG PET compared to 3.3 mm in CT images.

As already stated, various factors can influence image quality and therefore have an impact on the results of a radiomics model on CT as well as on FDG PET images [25–31]. Acquisition of FDG PET/CT was done on different machines with different parameter settings, which can affect the robustness of radiomic features [32]. Unfortunately, a small size of subcohorts with homogenous acquisition and reconstruction protocols prevented us from studying this effect in more detail or to apply correction methods, such as ComBat [33]. However, our dataset represent the real-life data heterogeneity. Retrospective nature of data collection together with rapid development of detector technology and reconstruction methods makes collection of large and homogenous datasets difficult. Therefore, we think that the recently introduced, specialized PET radiomics phantoms depicting heterogeneity of PET tracer uptake are key tools for robustness studies [32]. One further limitation of this study is the low patient number analyzed. In total, 505 CT features and 780 FDG PET features were analyzed for 36 patients in the training cohort. This leads to the risk of overfitting because we have little data for the number of analyzed variables [34]. Furthermore, we only used a multivariate logistic regression to assess the relation of radiomics results with patient outcome. As the prediction of PFS in MPM patients is a complex task with possible need for more predictor variables, a more complex model would eventually perform better in terms of outcome prediction. However, with our limited number of subjects, such an analysis was not possible and would require a much larger cohort—potentially requiring a multi-institutional project.

## Conclusions

We could show the prognostic potential of a FDG PET-based radiomics model for PFS in MPM patients on pre-treatment images. No CT-based model with sufficient discriminative power could be built. Radiomics could serve as a tool to aid decision support systems for treatment of patients with MPM—a malignancy whose curatively intended multimodal treatment can be challenging for patients, therefore, asking for a careful selection of appropriate candidates. However, further analysis with inclusion of more data in a multi-centric setting is recommended to validate the model.

## Supplementary information

**Additional file 1:** Stable FDG PET features.

**Additional file 2: Figure 1.** Cox regression model for volume for OS.

**Additional file 3.** Details on the multivariate Cox regression model for FDG PET radiomics.

## Data Availability

The datasets generated and analyzed during the current study are not publicly available but are available from the corresponding author on reasonable request.

## Abbreviations

MPM: Malignant pleural mesothelioma
PFS: Progression-free survival
OS: overall survival
FDG: Fluorine-18 fluorodeoxyglucose
HU: Hounsfield units
ICC: Intraclass correlation coefficient
SUV: Standard uptake value
MMPS: Multimodality Prognostic Score
