# Supplementary material for: FDG PET versus CT radiomics to predict outcome in malignant pleural mesothelioma patients
Source: EJNMMI Res. 2020 Jul 13;10:81. doi: 10.1186/s13550-020-00669-3 (PMC7359199; doi:10.1186/s13550-020-00669-3)
Supplement: Supplementary file 1 — Additional file 1: Stable FDG PET features. [file 13550_2020_669_MOESM1_ESM.pdf]

Stable shape features

Feature name	MPM
volume	✓
surface	
compactness 1	
compactness 2	
spherical disproportion	
sphericity	
asphericity	
surface to volume ratio	
median thickness	
standard deviation thickness	
euclidian distance	
major axis length	
minor axis length	
least axis length	
elongation	
flatness	
fractal dimension	
center of mass shift	

Table 1: Stable shape features, intraclass correlation coefficient >0.8. MPM – malignant pleural mesothelioma.

Stable intensity features

Feature name	MPM
metabolic tumor volume 20%	✓
metabolic tumor volume 30%	✓
metabolic tumor volume 40%	✓
metabolic tumor volume 50%	✓
metabolic tumor volume 60%	✓
metabolic tumor volume 70%	✓
mean	✓
standard deviation	✓
coefficient of variation	✓
skewness	
kurtosis	
variance	✓
median	✓
percentile 10th	
percentile 90th	✓
interquartile range	✓
range	✓
mean absolut deviation	✓
robust mean absolut deviation	✓
energy	✓
entropy	✓
root mean square	✓
uniformity	✓

Table 2: Stable intensity features, intraclass correlation coefficient >0.8. MPM – malignant pleural mesothelioma.

Stable texture features

Feature name	MPM
GLCM energy	✓
GLCM entropy	✓
GLCM contrast	✓
GLCM correlation	
GLCM homogeneity	✓
GLCM homogeneity normalized	
GLCM inverse difference	✓
GLCM inverse difference normalized	
GLCM variance	✓
GLCM sum of average	✓
GLCM sum of entropy	✓
GLCM sum of variance	✓
GLCM difference entropy	✓
GLCM difference variance	✓
GLCM information measures of correlation 1	
GLCM information measures of correlation 2	
GLCM maximal correlation coefficient	
GLCM joint maximum	✓
GLCM joint average	✓
GLCM difference average	✓
GLCM dissimilarity	✓
GLCM inverse variance	✓
GLCM autocorrelation	✓
GLCM cluster tendency	✓
GLCM cluster shade	✓
GLCM cluster prominence	✓
mGLCM energy	✓
mGLCM entropy	✓
mGLCM contrast	✓
mGLCM correlation	
mGLCM homogeneity	✓
mGLCM homogeneity normalized	
mGLCM inverse difference	✓
mGLCM inverse difference normalized	
mGLCM variance	✓
mGLCM sum of average	✓
mGLCM sum of entropy	✓
mGLCM sum of variance	✓
mGLCM difference entropy	✓

Feature name	MPM
mGLCM difference variance	✓
mGLCM information measures of correlation 1	
mGLCM information measures of correlation 2	
mGLCM maximal correlation coefficient	
mGLCM joint maximum	✓
mGLCM joint average	✓
mGLCM difference average	✓
mGLCM dissimilarity	✓
mGLCM inverse variance	✓
mGLCM autocorrelation	✓
mGLCM cluster tendency	✓
mGLCM cluster shade	✓
mGLCM cluster prominence	✓
NGTDM coarseness	
NGTDM contrast	✓
NGTDM busyness	
NGTDM complexity	✓
NGTDM strength	✓
GRLM grey level non-uniformity	
GRLM grey level non-uniformity normalized	✓
GRLM zone size non-uniformity	✓
GRLM zone size non-uniformity normalized	✓
GRLM short runs emphasis	✓
GRLM long runs emphasis	
GRLM low grey level run emphasis	
GRLM high grey level run emphasis	✓
GRLM short run low grey level emphasis	
GRLM short run high grey level emphasis	✓
GRLM long run low grey level emphasis	
GRLM long run high grey level emphasis	✓
GRLM run percentage	
GRLM grey level variance	✓
GRLM run length variance	
GRLM run entropy	✓
mGRLM grey level non-uniformity	
mGRLM grey level non-uniformity normalized	✓
mGRLM zone size non-uniformity	✓
mGRLM zone size non-uniformity normalized	✓
mGRLM short runs emphasis	✓
mGRLM long runs emphasis	
mGRLM low grey level run emphasis	

Feature name	MPM
mGRLM high grey level run emphasis	✓
mGRLM short run low grey level emphasis	
mGRLM short run high grey level emphasis	✓
mGRLM long run low grey level emphasis	
mGRLM long run high grey level emphasis	✓
mGRLM run percentage	
mGRLM grey level variance	✓
mGRLM run length variance	
mGRLM run entropy	✓
GLSZM grey level non-uniformity	✓
GLSZM grey level non-uniformity normalized	
GLSZM zone size non-uniformity	✓
GLSZM zone size non-uniformity normalized	✓
GLSZM small zone emphasis	
GLSZM large zone emphasis	
GLSZM low grey level zone emphasis	
GLSZM high grey level zone emphasis	✓
GLSZM small zone low grey level emphasis	
GLSZM small zone high grey level emphasis	✓
GLSZM large zone low grey level emphasis	
GLSZM large zone high grey level emphasis	
GLSZM zone percentage	✓
GLSZM grey level variance	✓
GLSZM zone size variance	
GLSZM zone size entropy	
GLDZM grey level non-uniformity	✓
GLDZM grey level non-uniformity normalized	
GLDZM zone size non-uniformity	✓
GLDZM zone size non-uniformity normalized	
GLDZM small distance emphasis	
GLDZM large distance emphasis	
GLDZM low grey level zone emphasis	
GLDZM high grey level zone emphasis	✓
GLDZM small distance low grey level emphasis	
GLDZM small distance high grey level emphasis	✓
GLDZM large distance low grey level emphasis	
GLDZM large distance high grey level emphasis	
GLDZM zone percentage	✓
GLDZM grey level variance	✓
GLDZM zone distance variance	
GLDZM zone distance entropy	✓
NGLDM grey level non-uniformity	

Feature name	MPM
NGLDM grey level non-uniformity normalized	✓
NGLDM dependence count non-uniformity	✓
NGLDM dependence count non-uniformity normalized	
NGLDM low dependence emphasis	✓
NGLDM high dependence emphasis	
NGLDM low grey level count emphasis	
NGLDM high grey level count emphasis	✓
NGLDM low dependence low grey level emphasis	
NGLDM low dependence high grey level emphasis	✓
NGLDM high dependence low grey level emphasis	
NGLDM high dependence high grey level emphasis	
NGLDM grey level variance	✓
NGLDM dependence count variance	
NGLDM dependence count entropy	✓
NGLDM dependence count energy	

Table 3: Stable texture features, intraclass correlation coefficient >0.8. MPM – malignant pleural mesothelioma, GLCM – the Gray Level Co-occurrence Matrix, mGLCM – the Gray Level Co-occurrence Matrix, NGTDM – the Neighborhood Gray Tone Difference Matrix, GLRM – the Gray Level Run Length Matrix, mGRLM – the merged Gray Level Run Length Matrix, GLSZM – the Gray Level Size Zone Matrix, GLDZM – the Gray Level Distance Zone Matrix, NGLDM – the Neighboring Gray Level Dependence Matrix.

Stable wavelet features

Feature name	MPM
HHH intensity mean	
HHH intensity standard deviation	✓
HHH intensity coefficient of variation	
HHH intensity skewness	
HHH intensity kurtosis	
HHH intensity variance	✓
HHH intensity median	
HHH intensity percentile 10th	✓
HHH intensity percentile 90th	✓
HHH intensity interquartile range	✓
HHH intensity range	✓
HHH intensity mean absolut deviation	✓
HHH intensity robust mean absolut deviation	✓
HHH intensity energy	✓
HHH intensity entropy	✓
HHH intensity root mean square	✓
HHH intensity uniformity	✓
HHH GLCM energy	✓
HHH GLCM entropy	✓
HHH GLCM contrast	✓
HHH GLCM correlation	✓
HHH GLCM homogeneity	✓
HHH GLCM homogeneity normalized	✓
HHH GLCM inverse difference	✓
HHH GLCM inverse difference normalized	✓
HHH GLCM variance	✓
HHH GLCM sum of average	✓
HHH GLCM sum of entropy	✓
HHH GLCM sum of variance	✓
HHH GLCM difference entropy	✓
HHH GLCM difference variance	✓
HHH GLCM information measures of correlation 1	
HHH GLCM information measures of correlation 2	✓
HHH GLCM maximal correlation coefficient	✓
HHH GLCM joint maximum	✓
HHH GLCM joint average	✓
HHH GLCM difference average	✓
HHH GLCM dissimilarity	✓

Feature name	MPM
HHH GLCM inverse variance	✓
HHH GLCM autocorrelation	✓
HHH GLCM cluster tendency	✓
HHH GLCM cluster shade	✓
HHH GLCM cluster prominence	✓
HHH mGLCM energy	✓
HHH mGLCM entropy	✓
HHH mGLCM contrast	✓
HHH mGLCM correlation	✓
HHH mGLCM homogeneity	✓
HHH mGLCM homogeneity normalized	✓
HHH mGLCM inverse difference	✓
HHH mGLCM inverse difference normalized	✓
HHH mGLCM variance	✓
HHH mGLCM sum of average	✓
HHH mGLCM sum of entropy	✓
HHH mGLCM sum of variance	✓
HHH mGLCM difference entropy	✓
HHH mGLCM difference variance	✓
HHH mGLCM information measures of correlation 1	✓
HHH mGLCM information measures of correlation 2	✓
HHH mGLCM maximal correlation coefficient	✓
HHH mGLCM joint maximum	✓
HHH mGLCM joint average	✓
HHH mGLCM difference average	✓
HHH mGLCM dissimilarity	✓
HHH mGLCM inverse variance	✓
HHH mGLCM autocorrelation	✓
HHH mGLCM cluster tendency	✓
HHH mGLCM cluster shade	✓
HHH mGLCM cluster prominence	✓
HHH NGTDM coarseness	✓
HHH NGTDM contrast	✓
HHH NGTDM busyness	
HHH NGTDM complexity	✓
HHH NGTDM strength	
HHH GRLM grey level non-uniformity	
HHH GRLM grey level non-uniformity normalized	✓
HHH GRLM zone size non-uniformity	✓
HHH GRLM zone size non-uniformity normalized	

Feature name	MPM
HHH GRLM short runs emphasis	
HHH GRLM long runs emphasis	
HHH GRLM low grey level run emphasis	✓
HHH GRLM high grey level run emphasis	✓
HHH GRLM short run low grey level emphasis	
HHH GRLM short run high grey level emphasis	✓
HHH GRLM long run low grey level emphasis	
HHH GRLM long run high grey level emphasis	
HHH GRLM run percentage	
HHH GRLM grey level variance	✓
HHH GRLM run length variance	
HHH GRLM run entropy	
HHH mGRLM grey level non-uniformity	
HHH mGRLM grey level non-uniformity normalized	✓
HHH mGRLM zone size non-uniformity	✓
HHH mGRLM zone size non-uniformity normalized	
HHH mGRLM short runs emphasis	
HHH mGRLM long runs emphasis	
HHH mGRLM low grey level run emphasis	✓
HHH mGRLM high grey level run emphasis	✓
HHH mGRLM short run low grey level emphasis	
HHH mGRLM short run high grey level emphasis	✓
HHH mGRLM long run low grey level emphasis	
HHH mGRLM long run high grey level emphasis	
HHH mGRLM run percentage	
HHH mGRLM grey level variance	✓
HHH mGRLM run length variance	
HHH mGRLM run entropy	
HHH GLSZM grey level non-uniformity	✓
HHH GLSZM grey level non-uniformity normalized	
HHH GLSZM zone size non-uniformity	✓
HHH GLSZM zone size non-uniformity normalized	
HHH GLSZM small zone emphasis	
HHH GLSZM large zone emphasis	
HHH GLSZM low grey level zone emphasis	✓
HHH GLSZM high grey level zone emphasis	✓
HHH GLSZM small zone low grey level emphasis	
HHH GLSZM small zone high grey level emphasis	
HHH GLSZM large zone low grey level emphasis	
HHH GLSZM large zone high grey level emphasis	
HHH GLSZM zone percentage	
HHH GLSZM grey level variance	✓
HHH GLSZM zone size variance	

Feature name	MPM
HHH GLSZM zone size entropy	
HHH GLDZM grey level non-uniformity	✓
HHH GLDZM grey level non-uniformity normalized	
HHH GLDZM zone size non-uniformity	✓
HHH GLDZM zone size non-uniformity normalized	
HHH GLDZM small distance emphasis	
HHH GLDZM large distance emphasis	
HHH GLDZM low grey level zone emphasis	✓
HHH GLDZM high grey level zone emphasis	✓
HHH GLDZM small distance low grey level emphasis	✓
HHH GLDZM small distance high grey level emphasis	✓
HHH GLDZM large distance low grey level emphasis	✓
HHH GLDZM large distance high grey level emphasis	✓
HHH GLDZM zone percentage	
HHH GLDZM grey level variance	✓
HHH GLDZM zone distance variance	
HHH GLDZM zone distance entropy	
HHH NGLDM grey level non-uniformity	
HHH NGLDM grey level non-uniformity normalized	✓
HHH NGLDM dependence count non-uniformity	
HHH NGLDM dependence count non-uniformity normalized	
HHH NGLDM low dependence emphasis	
HHH NGLDM high dependence emphasis	
HHH NGLDM low grey level count emphasis	✓
HHH NGLDM high grey level count emphasis	✓
HHH NGLDM low dependence low grey level emphasis	
HHH NGLDM low dependence high grey level emphasis	✓
HHH NGLDM high dependence low grey level emphasis	
HHH NGLDM high dependence high grey level emphasis	✓
HHH NGLDM grey level variance	✓
HHH NGLDM dependence count variance	
HHH NGLDM dependence count entropy	
HHH NGLDM dependence count energy	
HHL intensity mean	
HHL intensity standard deviation	✓
HHL intensity coefficient of variation	
HHL intensity skewness	
HHL intensity kurtosis	✓
HHL intensity variance	✓
HHL intensity median	
HHL intensity percentile 10th	✓
HHL intensity percentile 90th	✓

Feature name	MPM
HHL intensity interquartile range	✓
HHL intensity range	✓
HHL intensity mean absolut deviation	✓
HHL intensity robust mean absolut deviation	✓
HHL intensity energy	✓
HHL intensity entropy	
HHL intensity root mean square	✓
HHL intensity uniformity	
HHL GLCM energy	
HHL GLCM entropy	
HHL GLCM contrast	
HHL GLCM correlation	
HHL GLCM homogeneity	
HHL GLCM homogeneity normalized	
HHL GLCM inverse difference	
HHL GLCM inverse difference normalized	
HHL GLCM variance	
HHL GLCM sum of average	✓
HHL GLCM sum of entropy	
HHL GLCM sum of variance	
HHL GLCM difference entropy	✓
HHL GLCM difference variance	✓
HHL GLCM information measures of correlation 1	✓
HHL GLCM information measures of correlation 2	✓
HHL GLCM maximal correlation coefficient	✓
HHL GLCM joint maximum	
HHL GLCM joint average	✓
HHL GLCM difference average	
HHL GLCM dissimilarity	
HHL GLCM inverse variance	
HHL GLCM autocorrelation	✓
HHL GLCM cluster tendency	
HHL GLCM cluster shade	✓
HHL GLCM cluster prominence	✓
HHL mGLCM energy	
HHL mGLCM entropy	
HHL mGLCM contrast	
HHL mGLCM correlation	
HHL mGLCM homogeneity	
HHL mGLCM homogeneity normalized	
HHL mGLCM inverse difference	
HHL mGLCM inverse difference normalized	

Feature name	MPM
HHL mGLCM variance	
HHL mGLCM sum of average	✓
HHL mGLCM sum of entropy	
HHL mGLCM sum of variance	
HHL mGLCM difference entropy	✓
HHL mGLCM difference variance	✓
HHL mGLCM information measures of correlation 1	✓
HHL mGLCM information measures of correlation 2	✓
HHL mGLCM maximal correlation coefficient	✓
HHL mGLCM joint maximum	
HHL mGLCM joint average	✓
HHL mGLCM difference average	
HHL mGLCM dissimilarity	
HHL mGLCM inverse variance	
HHL mGLCM autocorrelation	✓
HHL mGLCM cluster tendency	
HHL mGLCM cluster shade	✓
HHL mGLCM cluster prominence	✓
HHL NGTDM coarseness	✓
HHL NGTDM contrast	
HHL NGTDM busyness	
HHL NGTDM complexity	✓
HHL NGTDM strength	
HHL GRLM grey level non-uniformity	
HHL GRLM grey level non-uniformity normalized	✓
HHL GRLM zone size non-uniformity	
HHL GRLM zone size non-uniformity normalized	
HHL GRLM short runs emphasis	
HHL GRLM long runs emphasis	
HHL GRLM low grey level run emphasis	✓
HHL GRLM high grey level run emphasis	✓
HHL GRLM short run low grey level emphasis	✓
HHL GRLM short run high grey level emphasis	✓
HHL GRLM long run low grey level emphasis	
HHL GRLM long run high grey level emphasis	
HHL GRLM run percentage	
HHL GRLM grey level variance	✓
HHL GRLM run length variance	
HHL GRLM run entropy	
HHL mGRLM grey level non-uniformity	
HHL mGRLM grey level non-uniformity normalized	✓
HHL mGRLM zone size non-uniformity	

Feature name	MPM
HHL mGRLM zone size non-uniformity normalized	
HHL mGRLM short runs emphasis	
HHL mGRLM long runs emphasis	
HHL mGRLM low grey level run emphasis	✓
HHL mGRLM high grey level run emphasis	✓
HHL mGRLM short run low grey level emphasis	✓
HHL mGRLM short run high grey level emphasis	✓
HHL mGRLM long run low grey level emphasis	
HHL mGRLM long run high grey level emphasis	
HHL mGRLM run percentage	
HHL mGRLM grey level variance	✓
HHL mGRLM run length variance	
HHL mGRLM run entropy	
HHL GLSZM grey level non-uniformity	
HHL GLSZM grey level non-uniformity normalized	
HHL GLSZM zone size non-uniformity	✓
HHL GLSZM zone size non-uniformity normalized	
HHL GLSZM small zone emphasis	
HHL GLSZM large zone emphasis	
HHL GLSZM low grey level zone emphasis	
HHL GLSZM high grey level zone emphasis	✓
HHL GLSZM small zone low grey level emphasis	
HHL GLSZM small zone high grey level emphasis	✓
HHL GLSZM large zone low grey level emphasis	
HHL GLSZM large zone high grey level emphasis	
HHL GLSZM zone percentage	
HHL GLSZM grey level variance	✓
HHL GLSZM zone size variance	
HHL GLSZM zone size entropy	✓
HHL GLDZM grey level non-uniformity	
HHL GLDZM grey level non-uniformity normalized	
HHL GLDZM zone size non-uniformity	
HHL GLDZM zone size non-uniformity normalized	
HHL GLDZM small distance emphasis	
HHL GLDZM large distance emphasis	
HHL GLDZM low grey level zone emphasis	
HHL GLDZM high grey level zone emphasis	✓
HHL GLDZM small distance low grey level emphasis	
HHL GLDZM small distance high grey level emphasis	✓
HHL GLDZM large distance low grey level emphasis	
HHL GLDZM large distance high grey level emphasis	
HHL GLDZM zone percentage	
HHL GLDZM grey level variance	✓

Feature name	MPM
HHL GLDZM zone distance variance	
HHL GLDZM zone distance entropy	✓
HHL NGLDM grey level non-uniformity	
HHL NGLDM grey level non-uniformity normalized	
HHL NGLDM dependence count non-uniformity	
HHL NGLDM dependence count non-uniformity normalized	
HHL NGLDM low dependence emphasis	
HHL NGLDM high dependence emphasis	
HHL NGLDM low grey level count emphasis	✓
HHL NGLDM high grey level count emphasis	✓
HHL NGLDM low dependence low grey level emphasis	
HHL NGLDM low dependence high grey level emphasis	✓
HHL NGLDM high dependence low grey level emphasis	
HHL NGLDM high dependence high grey level emphasis	✓
HHL NGLDM grey level variance	
HHL NGLDM dependence count variance	
HHL NGLDM dependence count entropy	
HHL NGLDM dependence count energy	
HLH intensity mean	
HLH intensity standard deviation	✓
HLH intensity coefficient of variation	
HLH intensity skewness	
HLH intensity kurtosis	
HLH intensity variance	✓
HLH intensity median	
HLH intensity percentile 10th	✓
HLH intensity percentile 90th	✓
HLH intensity interquartile range	✓
HLH intensity range	✓
HLH intensity mean absolut deviation	✓
HLH intensity robust mean absolut deviation	✓
HLH intensity energy	✓
HLH intensity entropy	✓
HLH intensity root mean square	✓
HLH intensity uniformity	✓
HLH GLCM energy	✓
HLH GLCM entropy	✓
HLH GLCM contrast	
HLH GLCM correlation	✓
HLH GLCM homogeneity	✓
HLH GLCM homogeneity normalized	
HLH GLCM inverse difference	✓

Feature name	MPM
HLH GLCM inverse difference normalized	✓
HLH GLCM variance	
HLH GLCM sum of average	✓
HLH GLCM sum of entropy	✓
HLH GLCM sum of variance	✓
HLH GLCM difference entropy	✓
HLH GLCM difference variance	✓
HLH GLCM information measures of correlation 1	✓
HLH GLCM information measures of correlation 2	✓
HLH GLCM maximal correlation coefficient	✓
HLH GLCM joint maximum	✓
HLH GLCM joint average	✓
HLH GLCM difference average	✓
HLH GLCM dissimilarity	✓
HLH GLCM inverse variance	✓
HLH GLCM autocorrelation	✓
HLH GLCM cluster tendency	✓
HLH GLCM cluster shade	
HLH GLCM cluster prominence	✓
HLH mGLCM energy	✓
HLH mGLCM entropy	✓
HLH mGLCM contrast	✓
HLH mGLCM correlation	
HLH mGLCM homogeneity	✓
HLH mGLCM homogeneity normalized	
HLH mGLCM inverse difference	✓
HLH mGLCM inverse difference normalized	✓
HLH mGLCM variance	✓
HLH mGLCM sum of average	✓
HLH mGLCM sum of entropy	✓
HLH mGLCM sum of variance	✓
HLH mGLCM difference entropy	✓
HLH mGLCM difference variance	✓
HLH mGLCM information measures of correlation 1	
HLH mGLCM information measures of correlation 2	✓
HLH mGLCM maximal correlation coefficient	✓
HLH mGLCM joint maximum	✓
HLH mGLCM joint average	✓
HLH mGLCM difference average	✓
HLH mGLCM dissimilarity	✓

Feature name	MPM
HLH mGLCM inverse variance	✓
HLH mGLCM autocorrelation	✓
HLH mGLCM cluster tendency	✓
HLH mGLCM cluster shade	
HLH mGLCM cluster prominence	✓
HLH NGTDM coarseness	
HLH NGTDM contrast	
HLH NGTDM busyness	
HLH NGTDM complexity	✓
HLH NGTDM strength	
HLH GRLM grey level non-uniformity	
HLH GRLM grey level non-uniformity normalized	✓
HLH GRLM zone size non-uniformity	✓
HLH GRLM zone size non-uniformity normalized	
HLH GRLM short runs emphasis	
HLH GRLM long runs emphasis	
HLH GRLM low grey level run emphasis	✓
HLH GRLM high grey level run emphasis	✓
HLH GRLM short run low grey level emphasis	✓
HLH GRLM short run high grey level emphasis	✓
HLH GRLM long run low grey level emphasis	
HLH GRLM long run high grey level emphasis	
HLH GRLM run percentage	
HLH GRLM grey level variance	✓
HLH GRLM run length variance	
HLH GRLM run entropy	
HLH mGRLM grey level non-uniformity	
HLH mGRLM grey level non-uniformity normalized	✓
HLH mGRLM zone size non-uniformity	✓
HLH mGRLM zone size non-uniformity normalized	
HLH mGRLM short runs emphasis	
HLH mGRLM long runs emphasis	
HLH mGRLM low grey level run emphasis	✓
HLH mGRLM high grey level run emphasis	✓
HLH mGRLM short run low grey level emphasis	✓
HLH mGRLM short run high grey level emphasis	✓
HLH mGRLM long run low grey level emphasis	
HLH mGRLM long run high grey level emphasis	
HLH mGRLM run percentage	
HLH mGRLM grey level variance	✓
HLH mGRLM run length variance	
HLH mGRLM run entropy	

Feature name	MPM
HLH GLSZM grey level non-uniformity	
HLH GLSZM grey level non-uniformity normalized	
HLH GLSZM zone size non-uniformity	✓
HLH GLSZM zone size non-uniformity normalized	
HLH GLSZM small zone emphasis	
HLH GLSZM large zone emphasis	
HLH GLSZM low grey level zone emphasis	
HLH GLSZM high grey level zone emphasis	✓
HLH GLSZM small zone low grey level emphasis	
HLH GLSZM small zone high grey level emphasis	✓
HLH GLSZM large zone low grey level emphasis	
HLH GLSZM large zone high grey level emphasis	
HLH GLSZM zone percentage	
HLH GLSZM grey level variance	✓
HLH GLSZM zone size variance	
HLH GLSZM zone size entropy	✓
HLH GLDZM grey level non-uniformity	
HLH GLDZM grey level non-uniformity normalized	
HLH GLDZM zone size non-uniformity	
HLH GLDZM zone size non-uniformity normalized	
HLH GLDZM small distance emphasis	
HLH GLDZM large distance emphasis	
HLH GLDZM low grey level zone emphasis	
HLH GLDZM high grey level zone emphasis	✓
HLH GLDZM small distance low grey level emphasis	
HLH GLDZM small distance high grey level emphasis	✓
HLH GLDZM large distance low grey level emphasis	
HLH GLDZM large distance high grey level emphasis	
HLH GLDZM zone percentage	
HLH GLDZM grey level variance	✓
HLH GLDZM zone distance variance	
HLH GLDZM zone distance entropy	✓
HLH NGLDM grey level non-uniformity	
HLH NGLDM grey level non-uniformity normalized	✓
HLH NGLDM dependence count non-uniformity	
HLH NGLDM dependence count non-uniformity normalized	
HLH NGLDM low dependence emphasis	✓
HLH NGLDM high dependence emphasis	
HLH NGLDM low grey level count emphasis	✓
HLH NGLDM high grey level count emphasis	✓
HLH NGLDM low dependence low grey level emphasis	
HLH NGLDM low dependence high grey level emphasis	✓
HLH NGLDM high dependence low grey level emphasis	

Feature name	MPM
HLH NGLDM high dependence high grey level emphasis	
HLH NGLDM grey level variance	✓
HLH NGLDM dependence count variance	
HLH NGLDM dependence count entropy	
HLH NGLDM dependence count energy	
HLL intensity mean	✓
HLL intensity standard deviation	✓
HLL intensity coefficient of variation	
HLL intensity skewness	
HLL intensity kurtosis	
HLL intensity variance	✓
HLL intensity median	
HLL intensity percentile 10th	
HLL intensity percentile 90th	✓
HLL intensity interquartile range	
HLL intensity range	✓
HLL intensity mean absolut deviation	✓
HLL intensity robust mean absolut deviation	
HLL intensity energy	✓
HLL intensity entropy	✓
HLL intensity root mean square	✓
HLL intensity uniformity	✓
HLL GLCM energy	✓
HLL GLCM entropy	✓
HLL GLCM contrast	✓
HLL GLCM correlation	
HLL GLCM homogeneity	✓
HLL GLCM homogeneity normalized	
HLL GLCM inverse difference	✓
HLL GLCM inverse difference normalized	
HLL GLCM variance	✓
HLL GLCM sum of average	✓
HLL GLCM sum of entropy	✓
HLL GLCM sum of variance	✓
HLL GLCM difference entropy	✓
HLL GLCM difference variance	✓
HLL GLCM information measures of correlation 1	✓
HLL GLCM information measures of correlation 2	✓
HLL GLCM maximal correlation coefficient	✓
HLL GLCM joint maximum	
HLL GLCM joint average	✓

Feature name	MPM
HLL GLCM difference average	✓
HLL GLCM dissimilarity	✓
HLL GLCM inverse variance	
HLL GLCM autocorrelation	
HLL GLCM cluster tendency	✓
HLL GLCM cluster shade	✓
HLL GLCM cluster prominence	✓
HLL mGLCM energy	✓
HLL mGLCM entropy	✓
HLL mGLCM contrast	✓
HLL mGLCM correlation	
HLL mGLCM homogeneity	✓
HLL mGLCM homogeneity normalized	
HLL mGLCM inverse difference	✓
HLL mGLCM inverse difference normalized	
HLL mGLCM variance	✓
HLL mGLCM sum of average	✓
HLL mGLCM sum of entropy	✓
HLL mGLCM sum of variance	✓
HLL mGLCM difference entropy	✓
HLL mGLCM difference variance	✓
HLL mGLCM information measures of correlation 1	
HLL mGLCM information measures of correlation 2	✓
HLL mGLCM maximal correlation coefficient	
HLL mGLCM joint maximum	
HLL mGLCM joint average	✓
HLL mGLCM difference average	✓
HLL mGLCM dissimilarity	✓
HLL mGLCM inverse variance	
HLL mGLCM autocorrelation	
HLL mGLCM cluster tendency	✓
HLL mGLCM cluster shade	✓
HLL mGLCM cluster prominence	✓
HLL NGTDM coarseness	
HLL NGTDM contrast	
HLL NGTDM busyness	
HLL NGTDM complexity	✓
HLL NGTDM strength	✓
HLL GRLM grey level non-uniformity	
HLL GRLM grey level non-uniformity normalized	✓
HLL GRLM zone size non-uniformity	✓

Feature name	MPM
HLL GRLM zone size non-uniformity normalized	
HLL GRLM short runs emphasis	
HLL GRLM long runs emphasis	
HLL GRLM low grey level run emphasis	✓
HLL GRLM high grey level run emphasis	
HLL GRLM short run low grey level emphasis	
HLL GRLM short run high grey level emphasis	✓
HLL GRLM long run low grey level emphasis	
HLL GRLM long run high grey level emphasis	
HLL GRLM run percentage	
HLL GRLM grey level variance	✓
HLL GRLM run length variance	
HLL GRLM run entropy	✓
HLL mGRLM grey level non-uniformity	
HLL mGRLM grey level non-uniformity normalized	✓
HLL mGRLM zone size non-uniformity	✓
HLL mGRLM zone size non-uniformity normalized	
HLL mGRLM short runs emphasis	
HLL mGRLM long runs emphasis	
HLL mGRLM low grey level run emphasis	✓
HLL mGRLM high grey level run emphasis	
HLL mGRLM short run low grey level emphasis	
HLL mGRLM short run high grey level emphasis	✓
HLL mGRLM long run low grey level emphasis	
HLL mGRLM long run high grey level emphasis	
HLL mGRLM run percentage	
HLL mGRLM grey level variance	✓
HLL mGRLM run length variance	
HLL mGRLM run entropy	✓
HLL GLSZM grey level non-uniformity	
HLL GLSZM grey level non-uniformity normalized	
HLL GLSZM zone size non-uniformity	✓
HLL GLSZM zone size non-uniformity normalized	
HLL GLSZM small zone emphasis	
HLL GLSZM large zone emphasis	
HLL GLSZM low grey level zone emphasis	
HLL GLSZM high grey level zone emphasis	
HLL GLSZM small zone low grey level emphasis	
HLL GLSZM small zone high grey level emphasis	
HLL GLSZM large zone low grey level emphasis	
HLL GLSZM large zone high grey level emphasis	
HLL GLSZM zone percentage	
HLL GLSZM grey level variance	✓

Feature name	MPM
HLL GLSZM zone size variance	
HLL GLSZM zone size entropy	✓
HLL GLDZM grey level non-uniformity	
HLL GLDZM grey level non-uniformity normalized	
HLL GLDZM zone size non-uniformity	✓
HLL GLDZM zone size non-uniformity normalized	
HLL GLDZM small distance emphasis	
HLL GLDZM large distance emphasis	
HLL GLDZM low grey level zone emphasis	
HLL GLDZM high grey level zone emphasis	
HLL GLDZM small distance low grey level emphasis	
HLL GLDZM small distance high grey level emphasis	
HLL GLDZM large distance low grey level emphasis	
HLL GLDZM large distance high grey level emphasis	
HLL GLDZM zone percentage	
HLL GLDZM grey level variance	✓
HLL GLDZM zone distance variance	
HLL GLDZM zone distance entropy	✓
HLL NGLDM grey level non-uniformity	
HLL NGLDM grey level non-uniformity normalized	✓
HLL NGLDM dependence count non-uniformity	✓
HLL NGLDM dependence count non-uniformity normalized	
HLL NGLDM low dependence emphasis	✓
HLL NGLDM high dependence emphasis	
HLL NGLDM low grey level count emphasis	✓
HLL NGLDM high grey level count emphasis	
HLL NGLDM low dependence low grey level emphasis	
HLL NGLDM low dependence high grey level emphasis	
HLL NGLDM high dependence low grey level emphasis	
HLL NGLDM high dependence high grey level emphasis	
HLL NGLDM grey level variance	✓
HLL NGLDM dependence count variance	
HLL NGLDM dependence count entropy	✓
HLL NGLDM dependence count energy	
LHH intensity mean	
LHH intensity standard deviation	✓
LHH intensity coefficient of variation	
LHH intensity skewness	
LHH intensity kurtosis	
LHH intensity variance	✓
LHH intensity median	
LHH intensity percentile 10th	✓
LHH intensity percentile 90th	✓

Feature name	MPM
LHH intensity interquartile range	✓
LHH intensity range	✓
LHH intensity mean absolut deviation	✓
LHH intensity robust mean absolut deviation	✓
LHH intensity energy	✓
LHH intensity entropy	✓
LHH intensity root mean square	✓
LHH intensity uniformity	✓
LHH GLCM energy	✓
LHH GLCM entropy	✓
LHH GLCM contrast	✓
LHH GLCM correlation	
LHH GLCM homogeneity	✓
LHH GLCM homogeneity normalized	
LHH GLCM inverse difference	✓
LHH GLCM inverse difference normalized	✓
LHH GLCM variance	✓
LHH GLCM sum of average	✓
LHH GLCM sum of entropy	✓
LHH GLCM sum of variance	✓
LHH GLCM difference entropy	✓
LHH GLCM difference variance	✓
LHH GLCM information measures of correlation 1	✓
LHH GLCM information measures of correlation 2	✓
LHH GLCM maximal correlation coefficient	✓
LHH GLCM joint maximum	✓
LHH GLCM joint average	✓
LHH GLCM difference average	✓
LHH GLCM dissimilarity	✓
LHH GLCM inverse variance	✓
LHH GLCM autocorrelation	✓
LHH GLCM cluster tendency	✓
LHH GLCM cluster shade	✓
LHH GLCM cluster prominence	✓
LHH mGLCM energy	✓
LHH mGLCM entropy	✓
LHH mGLCM contrast	✓
LHH mGLCM correlation	
LHH mGLCM homogeneity	✓
LHH mGLCM homogeneity normalized	

Feature name	MPM
LHH mGLCM inverse difference	✓
LHH mGLCM inverse difference normalized	✓
LHH mGLCM variance	✓
LHH mGLCM sum of average	✓
LHH mGLCM sum of entropy	✓
LHH mGLCM sum of variance	✓
LHH mGLCM difference entropy	✓
LHH mGLCM difference variance	✓
LHH mGLCM information measures of correlation 1	
LHH mGLCM information measures of correlation 2	✓
LHH mGLCM maximal correlation coefficient	✓
LHH mGLCM joint maximum	✓
LHH mGLCM joint average	✓
LHH mGLCM difference average	✓
LHH mGLCM dissimilarity	✓
LHH mGLCM inverse variance	✓
LHH mGLCM autocorrelation	✓
LHH mGLCM cluster tendency	✓
LHH mGLCM cluster shade	✓
LHH mGLCM cluster prominence	✓
LHH NGTDM coarseness	✓
LHH NGTDM contrast	
LHH NGTDM busyness	
LHH NGTDM complexity	✓
LHH NGTDM strength	
LHH GRLM grey level non-uniformity	
LHH GRLM grey level non-uniformity normalized	✓
LHH GRLM zone size non-uniformity	
LHH GRLM zone size non-uniformity normalized	
LHH GRLM short runs emphasis	
LHH GRLM long runs emphasis	
LHH GRLM low grey level run emphasis	✓
LHH GRLM high grey level run emphasis	✓
LHH GRLM short run low grey level emphasis	
LHH GRLM short run high grey level emphasis	✓
LHH GRLM long run low grey level emphasis	
LHH GRLM long run high grey level emphasis	
LHH GRLM run percentage	
LHH GRLM grey level variance	✓
LHH GRLM run length variance	
LHH GRLM run entropy	

Feature name	MPM
LHH mGRLM grey level non-uniformity	
LHH mGRLM grey level non-uniformity normalized	✓
LHH mGRLM zone size non-uniformity	
LHH mGRLM zone size non-uniformity normalized	
LHH mGRLM short runs emphasis	
LHH mGRLM long runs emphasis	
LHH mGRLM low grey level run emphasis	✓
LHH mGRLM high grey level run emphasis	✓
LHH mGRLM short run low grey level emphasis	✓
LHH mGRLM short run high grey level emphasis	✓
LHH mGRLM long run low grey level emphasis	
LHH mGRLM long run high grey level emphasis	
LHH mGRLM run percentage	
LHH mGRLM grey level variance	✓
LHH mGRLM run length variance	
LHH mGRLM run entropy	
LHH GLSZM grey level non-uniformity	
LHH GLSZM grey level non-uniformity normalized	✓
LHH GLSZM zone size non-uniformity	✓
LHH GLSZM zone size non-uniformity normalized	
LHH GLSZM small zone emphasis	
LHH GLSZM large zone emphasis	
LHH GLSZM low grey level zone emphasis	✓
LHH GLSZM high grey level zone emphasis	✓
LHH GLSZM small zone low grey level emphasis	
LHH GLSZM small zone high grey level emphasis	✓
LHH GLSZM large zone low grey level emphasis	
LHH GLSZM large zone high grey level emphasis	
LHH GLSZM zone percentage	
LHH GLSZM grey level variance	✓
LHH GLSZM zone size variance	
LHH GLSZM zone size entropy	✓
LHH GLDZM grey level non-uniformity	
LHH GLDZM grey level non-uniformity normalized	✓
LHH GLDZM zone size non-uniformity	
LHH GLDZM zone size non-uniformity normalized	
LHH GLDZM small distance emphasis	
LHH GLDZM large distance emphasis	
LHH GLDZM low grey level zone emphasis	✓
LHH GLDZM high grey level zone emphasis	✓
LHH GLDZM small distance low grey level emphasis	✓
LHH GLDZM small distance high grey level emphasis	✓

Feature name	MPM
LHH GLDZM large distance low grey level emphasis	
LHH GLDZM large distance high grey level emphasis	
LHH GLDZM zone percentage	
LHH GLDZM grey level variance	✓
LHH GLDZM zone distance variance	
LHH GLDZM zone distance entropy	✓
LHH NGLDM grey level non-uniformity	
LHH NGLDM grey level non-uniformity normalized	✓
LHH NGLDM dependence count non-uniformity	
LHH NGLDM dependence count non-uniformity normalized	
LHH NGLDM low dependence emphasis	
LHH NGLDM high dependence emphasis	
LHH NGLDM low grey level count emphasis	✓
LHH NGLDM high grey level count emphasis	✓
LHH NGLDM low dependence low grey level emphasis	
LHH NGLDM low dependence high grey level emphasis	✓
LHH NGLDM high dependence low grey level emphasis	
LHH NGLDM high dependence high grey level emphasis	✓
LHH NGLDM grey level variance	✓
LHH NGLDM dependence count variance	
LHH NGLDM dependence count entropy	
LHH NGLDM dependence count energy	
LHL intensity mean	
LHL intensity standard deviation	✓
LHL intensity coefficient of variation	
LHL intensity skewness	
LHL intensity kurtosis	✓
LHL intensity variance	✓
LHL intensity median	
LHL intensity percentile 10th	✓
LHL intensity percentile 90th	✓
LHL intensity interquartile range	✓
LHL intensity range	✓
LHL intensity mean absolut deviation	✓
LHL intensity robust mean absolut deviation	✓
LHL intensity energy	✓
LHL intensity entropy	✓
LHL intensity root mean square	✓
LHL intensity uniformity	✓
LHL GLCM energy	
LHL GLCM entropy	✓

Feature name	MPM
LHL GLCM contrast	✓
LHL GLCM correlation	
LHL GLCM homogeneity	✓
LHL GLCM homogeneity normalized	
LHL GLCM inverse difference	✓
LHL GLCM inverse difference normalized	
LHL GLCM variance	✓
LHL GLCM sum of average	✓
LHL GLCM sum of entropy	✓
LHL GLCM sum of variance	✓
LHL GLCM difference entropy	✓
LHL GLCM difference variance	✓
LHL GLCM information measures of correlation 1	
LHL GLCM information measures of correlation 2	✓
LHL GLCM maximal correlation coefficient	✓
LHL GLCM joint maximum	✓
LHL GLCM joint average	✓
LHL GLCM difference average	✓
LHL GLCM dissimilarity	✓
LHL GLCM inverse variance	
LHL GLCM autocorrelation	✓
LHL GLCM cluster tendency	✓
LHL GLCM cluster shade	✓
LHL GLCM cluster prominence	✓
LHL mGLCM energy	
LHL mGLCM entropy	✓
LHL mGLCM contrast	✓
LHL mGLCM correlation	
LHL mGLCM homogeneity	✓
LHL mGLCM homogeneity normalized	
LHL mGLCM inverse difference	✓
LHL mGLCM inverse difference normalized	
LHL mGLCM variance	✓
LHL mGLCM sum of average	✓
LHL mGLCM sum of entropy	✓
LHL mGLCM sum of variance	✓
LHL mGLCM difference entropy	✓
LHL mGLCM difference variance	✓
LHL mGLCM information measures of correlation 1	
LHL mGLCM information measures of correlation 2	✓

Feature name	MPM
LHL mGLCM maximal correlation coefficient	✓
LHL mGLCM joint maximum	✓
LHL mGLCM joint average	✓
LHL mGLCM difference average	✓
LHL mGLCM dissimilarity	✓
LHL mGLCM inverse variance	
LHL mGLCM autocorrelation	✓
LHL mGLCM cluster tendency	✓
LHL mGLCM cluster shade	✓
LHL mGLCM cluster prominence	✓
LHL NGTDM coarseness	✓
LHL NGTDM contrast	
LHL NGTDM busyness	
LHL NGTDM complexity	✓
LHL NGTDM strength	
LHL GRLM grey level non-uniformity	
LHL GRLM grey level non-uniformity normalized	✓
LHL GRLM zone size non-uniformity	✓
LHL GRLM zone size non-uniformity normalized	
LHL GRLM short runs emphasis	
LHL GRLM long runs emphasis	
LHL GRLM low grey level run emphasis	✓
LHL GRLM high grey level run emphasis	✓
LHL GRLM short run low grey level emphasis	✓
LHL GRLM short run high grey level emphasis	✓
LHL GRLM long run low grey level emphasis	
LHL GRLM long run high grey level emphasis	✓
LHL GRLM run percentage	
LHL GRLM grey level variance	✓
LHL GRLM run length variance	
LHL GRLM run entropy	
LHL mGRLM grey level non-uniformity	
LHL mGRLM grey level non-uniformity normalized	✓
LHL mGRLM zone size non-uniformity	✓
LHL mGRLM zone size non-uniformity normalized	
LHL mGRLM short runs emphasis	
LHL mGRLM long runs emphasis	
LHL mGRLM low grey level run emphasis	✓
LHL mGRLM high grey level run emphasis	✓
LHL mGRLM short run low grey level emphasis	✓
LHL mGRLM short run high grey level emphasis	✓

Feature name	MPM
LHL mGRLM long run low grey level emphasis	
LHL mGRLM long run high grey level emphasis	✓
LHL mGRLM run percentage	
LHL mGRLM grey level variance	✓
LHL mGRLM run length variance	
LHL mGRLM run entropy	
LHL GLSZM grey level non-uniformity	
LHL GLSZM grey level non-uniformity normalized	✓
LHL GLSZM zone size non-uniformity	✓
LHL GLSZM zone size non-uniformity normalized	
LHL GLSZM small zone emphasis	✓
LHL GLSZM large zone emphasis	
LHL GLSZM low grey level zone emphasis	✓
LHL GLSZM high grey level zone emphasis	✓
LHL GLSZM small zone low grey level emphasis	
LHL GLSZM small zone high grey level emphasis	✓
LHL GLSZM large zone low grey level emphasis	
LHL GLSZM large zone high grey level emphasis	
LHL GLSZM zone percentage	
LHL GLSZM grey level variance	✓
LHL GLSZM zone size variance	
LHL GLSZM zone size entropy	✓
LHL GLDZM grey level non-uniformity	
LHL GLDZM grey level non-uniformity normalized	✓
LHL GLDZM zone size non-uniformity	✓
LHL GLDZM zone size non-uniformity normalized	
LHL GLDZM small distance emphasis	
LHL GLDZM large distance emphasis	
LHL GLDZM low grey level zone emphasis	✓
LHL GLDZM high grey level zone emphasis	✓
LHL GLDZM small distance low grey level emphasis	✓
LHL GLDZM small distance high grey level emphasis	✓
LHL GLDZM large distance low grey level emphasis	✓
LHL GLDZM large distance high grey level emphasis	✓
LHL GLDZM zone percentage	
LHL GLDZM grey level variance	✓
LHL GLDZM zone distance variance	
LHL GLDZM zone distance entropy	✓
LHL NGLDM grey level non-uniformity	
LHL NGLDM grey level non-uniformity normalized	✓
LHL NGLDM dependence count non-uniformity	
LHL NGLDM dependence count non-uniformity normalized	

Feature name	MPM
LHL NGLDM low dependence emphasis	✓
LHL NGLDM high dependence emphasis	
LHL NGLDM low grey level count emphasis	✓
LHL NGLDM high grey level count emphasis	✓
LHL NGLDM low dependence low grey level emphasis	
LHL NGLDM low dependence high grey level emphasis	✓
LHL NGLDM high dependence low grey level emphasis	
LHL NGLDM high dependence high grey level emphasis	
LHL NGLDM grey level variance	✓
LHL NGLDM dependence count variance	
LHL NGLDM dependence count entropy	✓
LHL NGLDM dependence count energy	
LLH intensity mean	
LLH intensity standard deviation	✓
LLH intensity coefficient of variation	
LLH intensity skewness	
LLH intensity kurtosis	
LLH intensity variance	✓
LLH intensity median	
LLH intensity percentile 10th	✓
LLH intensity percentile 90th	✓
LLH intensity interquartile range	✓
LLH intensity range	✓
LLH intensity mean absolut deviation	✓
LLH intensity robust mean absolut deviation	✓
LLH intensity energy	✓
LLH intensity entropy	✓
LLH intensity root mean square	✓
LLH intensity uniformity	✓
LLH GLCM energy	✓
LLH GLCM entropy	✓
LLH GLCM contrast	✓
LLH GLCM correlation	
LLH GLCM homogeneity	✓
LLH GLCM homogeneity normalized	
LLH GLCM inverse difference	✓
LLH GLCM inverse difference normalized	
LLH GLCM variance	✓
LLH GLCM sum of average	✓
LLH GLCM sum of entropy	✓
LLH GLCM sum of variance	✓

Feature name	MPM
LLH GLCM difference entropy	✓
LLH GLCM difference variance	✓
LLH GLCM information measures of correlation 1	
LLH GLCM information measures of correlation 2	✓
LLH GLCM maximal correlation coefficient	
LLH GLCM joint maximum	✓
LLH GLCM joint average	✓
LLH GLCM difference average	✓
LLH GLCM dissimilarity	✓
LLH GLCM inverse variance	
LLH GLCM autocorrelation	
LLH GLCM cluster tendency	✓
LLH GLCM cluster shade	
LLH GLCM cluster prominence	✓
LLH mGLCM energy	✓
LLH mGLCM entropy	✓
LLH mGLCM contrast	✓
LLH mGLCM correlation	
LLH mGLCM homogeneity	✓
LLH mGLCM homogeneity normalized	
LLH mGLCM inverse difference	✓
LLH mGLCM inverse difference normalized	
LLH mGLCM variance	✓
LLH mGLCM sum of average	✓
LLH mGLCM sum of entropy	✓
LLH mGLCM sum of variance	✓
LLH mGLCM difference entropy	✓
LLH mGLCM difference variance	✓
LLH mGLCM information measures of correlation 1	
LLH mGLCM information measures of correlation 2	✓
LLH mGLCM maximal correlation coefficient	
LLH mGLCM joint maximum	✓
LLH mGLCM joint average	✓
LLH mGLCM difference average	✓
LLH mGLCM dissimilarity	✓
LLH mGLCM inverse variance	
LLH mGLCM autocorrelation	
LLH mGLCM cluster tendency	✓
LLH mGLCM cluster shade	
LLH mGLCM cluster prominence	✓
LLH NGTDM coarseness	

Feature name	MPM
LLH NGTDM contrast	
LLH NGTDM busyness	
LLH NGTDM complexity	✓
LLH NGTDM strength	✓
LLH GRLM grey level non-uniformity	
LLH GRLM grey level non-uniformity normalized	✓
LLH GRLM zone size non-uniformity	✓
LLH GRLM zone size non-uniformity normalized	
LLH GRLM short runs emphasis	
LLH GRLM long runs emphasis	
LLH GRLM low grey level run emphasis	✓
LLH GRLM high grey level run emphasis	
LLH GRLM short run low grey level emphasis	✓
LLH GRLM short run high grey level emphasis	✓
LLH GRLM long run low grey level emphasis	
LLH GRLM long run high grey level emphasis	
LLH GRLM run percentage	
LLH GRLM grey level variance	✓
LLH GRLM run length variance	
LLH GRLM run entropy	
LLH mGRLM grey level non-uniformity	
LLH mGRLM grey level non-uniformity normalized	✓
LLH mGRLM zone size non-uniformity	✓
LLH mGRLM zone size non-uniformity normalized	
LLH mGRLM short runs emphasis	
LLH mGRLM long runs emphasis	
LLH mGRLM low grey level run emphasis	✓
LLH mGRLM high grey level run emphasis	
LLH mGRLM short run low grey level emphasis	✓
LLH mGRLM short run high grey level emphasis	✓
LLH mGRLM long run low grey level emphasis	
LLH mGRLM long run high grey level emphasis	
LLH mGRLM run percentage	
LLH mGRLM grey level variance	✓
LLH mGRLM run length variance	
LLH mGRLM run entropy	
LLH GLSZM grey level non-uniformity	
LLH GLSZM grey level non-uniformity normalized	✓
LLH GLSZM zone size non-uniformity	✓
LLH GLSZM zone size non-uniformity normalized	
LLH GLSZM small zone emphasis	
LLH GLSZM large zone emphasis	

Feature name	MPM
LLH GLSZM low grey level zone emphasis	
LLH GLSZM high grey level zone emphasis	
LLH GLSZM small zone low grey level emphasis	
LLH GLSZM small zone high grey level emphasis	
LLH GLSZM large zone low grey level emphasis	
LLH GLSZM large zone high grey level emphasis	
LLH GLSZM zone percentage	
LLH GLSZM grey level variance	✓
LLH GLSZM zone size variance	
LLH GLSZM zone size entropy	✓
LLH GLDZM grey level non-uniformity	
LLH GLDZM grey level non-uniformity normalized	✓
LLH GLDZM zone size non-uniformity	✓
LLH GLDZM zone size non-uniformity normalized	
LLH GLDZM small distance emphasis	
LLH GLDZM large distance emphasis	
LLH GLDZM low grey level zone emphasis	
LLH GLDZM high grey level zone emphasis	
LLH GLDZM small distance low grey level emphasis	
LLH GLDZM small distance high grey level emphasis	✓
LLH GLDZM large distance low grey level emphasis	
LLH GLDZM large distance high grey level emphasis	
LLH GLDZM zone percentage	
LLH GLDZM grey level variance	✓
LLH GLDZM zone distance variance	
LLH GLDZM zone distance entropy	✓
LLH NGLDM grey level non-uniformity	
LLH NGLDM grey level non-uniformity normalized	✓
LLH NGLDM dependence count non-uniformity	
LLH NGLDM dependence count non-uniformity normalized	
LLH NGLDM low dependence emphasis	✓
LLH NGLDM high dependence emphasis	
LLH NGLDM low grey level count emphasis	✓
LLH NGLDM high grey level count emphasis	
LLH NGLDM low dependence low grey level emphasis	
LLH NGLDM low dependence high grey level emphasis	✓
LLH NGLDM high dependence low grey level emphasis	
LLH NGLDM high dependence high grey level emphasis	
LLH NGLDM grey level variance	✓
LLH NGLDM dependence count variance	
LLH NGLDM dependence count entropy	
LLH NGLDM dependence count energy	
LLL intensity mean	✓

Feature name	MPM
LLL intensity standard deviation	✓
LLL intensity coefficient of variation	✓
LLL intensity skewness	
LLL intensity kurtosis	
LLL intensity variance	✓
LLL intensity median	✓
LLL intensity percentile 10th	
LLL intensity percentile 90th	✓
LLL intensity interquartile range	✓
LLL intensity range	✓
LLL intensity mean absolut deviation	✓
LLL intensity robust mean absolut deviation	✓
LLL intensity energy	✓
LLL intensity entropy	✓
LLL intensity root mean square	✓
LLL intensity uniformity	✓
LLL GLCM energy	
LLL GLCM entropy	✓
LLL GLCM contrast	✓
LLL GLCM correlation	
LLL GLCM homogeneity	✓
LLL GLCM homogeneity normalized	
LLL GLCM inverse difference	✓
LLL GLCM inverse difference normalized	
LLL GLCM variance	✓
LLL GLCM sum of average	✓
LLL GLCM sum of entropy	✓
LLL GLCM sum of variance	✓
LLL GLCM difference entropy	✓
LLL GLCM difference variance	✓
LLL GLCM information measures of correlation 1	
LLL GLCM information measures of correlation 2	✓
LLL GLCM maximal correlation coefficient	
LLL GLCM joint maximum	
LLL GLCM joint average	✓
LLL GLCM difference average	✓
LLL GLCM dissimilarity	✓
LLL GLCM inverse variance	✓
LLL GLCM autocorrelation	✓
LLL GLCM cluster tendency	✓

Feature name	MPM
LLL GLCM cluster shade	✓
LLL GLCM cluster prominence	✓
LLL mGLCM energy	✓
LLL mGLCM entropy	✓
LLL mGLCM contrast	✓
LLL mGLCM correlation	
LLL mGLCM homogeneity	✓
LLL mGLCM homogeneity normalized	
LLL mGLCM inverse difference	✓
LLL mGLCM inverse difference normalized	
LLL mGLCM variance	✓
LLL mGLCM sum of average	✓
LLL mGLCM sum of entropy	✓
LLL mGLCM sum of variance	✓
LLL mGLCM difference entropy	✓
LLL mGLCM difference variance	✓
LLL mGLCM information measures of correlation 1	
LLL mGLCM information measures of correlation 2	
LLL mGLCM maximal correlation coefficient	
LLL mGLCM joint maximum	
LLL mGLCM joint average	✓
LLL mGLCM difference average	✓
LLL mGLCM dissimilarity	✓
LLL mGLCM inverse variance	✓
LLL mGLCM autocorrelation	✓
LLL mGLCM cluster tendency	✓
LLL mGLCM cluster shade	✓
LLL mGLCM cluster prominence	✓
LLL NGTDM coarseness	
LLL NGTDM contrast	
LLL NGTDM busyness	
LLL NGTDM complexity	✓
LLL NGTDM strength	✓
LLL GRLM grey level non-uniformity	
LLL GRLM grey level non-uniformity normalized	✓
LLL GRLM zone size non-uniformity	✓
LLL GRLM zone size non-uniformity normalized	✓
LLL GRLM short runs emphasis	✓
LLL GRLM long runs emphasis	
LLL GRLM low grey level run emphasis	✓
LLL GRLM high grey level run emphasis	✓

Feature name	MPM
LLL GRLM short run low grey level emphasis	
LLL GRLM short run high grey level emphasis	✓
LLL GRLM long run low grey level emphasis	
LLL GRLM long run high grey level emphasis	✓
LLL GRLM run percentage	
LLL GRLM grey level variance	✓
LLL GRLM run length variance	
LLL GRLM run entropy	✓
LLL mGRLM grey level non-uniformity	
LLL mGRLM grey level non-uniformity normalized	✓
LLL mGRLM zone size non-uniformity	✓
LLL mGRLM zone size non-uniformity normalized	✓
LLL mGRLM short runs emphasis	
LLL mGRLM long runs emphasis	
LLL mGRLM low grey level run emphasis	✓
LLL mGRLM high grey level run emphasis	✓
LLL mGRLM short run low grey level emphasis	
LLL mGRLM short run high grey level emphasis	✓
LLL mGRLM long run low grey level emphasis	
LLL mGRLM long run high grey level emphasis	✓
LLL mGRLM run percentage	
LLL mGRLM grey level variance	✓
LLL mGRLM run length variance	
LLL mGRLM run entropy	✓
LLL GLSZM grey level non-uniformity	✓
LLL GLSZM grey level non-uniformity normalized	✓
LLL GLSZM zone size non-uniformity	✓
LLL GLSZM zone size non-uniformity normalized	
LLL GLSZM small zone emphasis	
LLL GLSZM large zone emphasis	
LLL GLSZM low grey level zone emphasis	
LLL GLSZM high grey level zone emphasis	✓
LLL GLSZM small zone low grey level emphasis	
LLL GLSZM small zone high grey level emphasis	✓
LLL GLSZM large zone low grey level emphasis	
LLL GLSZM large zone high grey level emphasis	
LLL GLSZM zone percentage	✓
LLL GLSZM grey level variance	✓
LLL GLSZM zone size variance	
LLL GLSZM zone size entropy	✓
LLL GLDZM grey level non-uniformity	✓

Feature name	MPM
LLL GLDZM grey level non-uniformity normalized	✓
LLL GLDZM zone size non-uniformity	✓
LLL GLDZM zone size non-uniformity normalized	
LLL GLDZM small distance emphasis	
LLL GLDZM large distance emphasis	
LLL GLDZM low grey level zone emphasis	
LLL GLDZM high grey level zone emphasis	✓
LLL GLDZM small distance low grey level emphasis	
LLL GLDZM small distance high grey level emphasis	✓
LLL GLDZM large distance low grey level emphasis	
LLL GLDZM large distance high grey level emphasis	✓
LLL GLDZM zone percentage	✓
LLL GLDZM grey level variance	✓
LLL GLDZM zone distance variance	
LLL GLDZM zone distance entropy	✓
LLL NGLDM grey level non-uniformity	
LLL NGLDM grey level non-uniformity normalized	✓
LLL NGLDM dependence count non-uniformity	✓
LLL NGLDM dependence count non-uniformity normalized	
LLL NGLDM low dependence emphasis	✓
LLL NGLDM high dependence emphasis	
LLL NGLDM low grey level count emphasis	✓
LLL NGLDM high grey level count emphasis	✓
LLL NGLDM low dependence low grey level emphasis	
LLL NGLDM low dependence high grey level emphasis	✓
LLL NGLDM high dependence low grey level emphasis	
LLL NGLDM high dependence high grey level emphasis	
LLL NGLDM grey level variance	✓
LLL NGLDM dependence count variance	
LLL NGLDM dependence count entropy	
LLL NGLDM dependence count energy	

Table 4: Stable wavelet features, intraclass correlation coefficient >0.8. MPM – malignant pleural mesothelioma, GLCM – the Gray Level Co-occurrence Matrix, mGLCM – the Gray Level Co-occurrence Matrix, NGTDM – the Neighborhood Gray Tone Difference Matrix, GLRM – the Gray Level Run Length Matrix, mGRLM – the merged Gray Level Run Length Matrix, GLSZM – the Gray Level Size Zone Matrix, GLDZM – the Gray Level Distance Zone Matrix, NGLDM – the Neighboring Gray Level Dependence Matrix, H – high–pass filter, L – low–pass filter
