# Supplementary figures and images for: FDG PET versus CT radiomics to predict outcome in malignant pleural mesothelioma patients
Source: EJNMMI Res. 2020 Jul 13;10:81. doi: 10.1186/s13550-020-00669-3 (PMC7359199; doi:10.1186/s13550-020-00669-3)

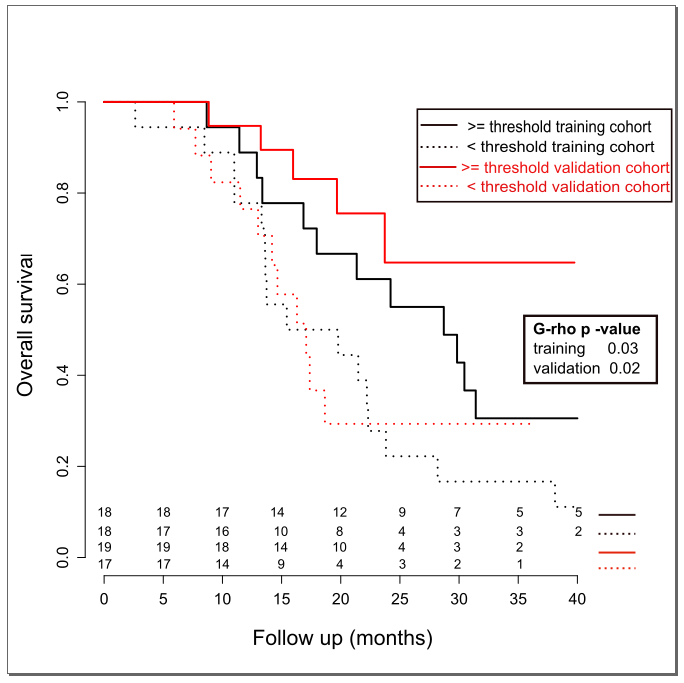


Figure 1. Cox regression model for Volume for OS

Supplement: Supplementary file 2 — Additional file 2: Figure 1. Cox regression model for volume for OS. [file 13550_2020_669_MOESM2_ESM.docx]
