# Supplementary material for: FDG PET versus CT radiomics to predict outcome in malignant pleural mesothelioma patients
Source: EJNMMI Res. 2020 Jul 13;10:81. doi: 10.1186/s13550-020-00669-3 (PMC7359199; doi:10.1186/s13550-020-00669-3)

**Details on the multivariate Cox regression model for FDG-PET radiomics**

*Model parameters*

| **Feature name** | **Coefficient** | **p-value** |
| --- | --- | --- |
| HLH GLCM maximal correlation coefficient | 11.45 | 0.006 |
| HLH intensity range | -1.93 | 0.021 |
| HLH GLSZM high grey level zone emphasis | 0.063 | 0.049 |

*Estimation of the base hazard function*

The base hazard for this cohort can be estimated using the following function

$$\ln\left( H_{0}\left( t \right) \right)= 2.72-\frac{35.15}{t}$$

This model explains 88% of data variance (R^2^ = 0.88). It describes the curves well until the timepoint of 20 months. For later timepoints, the model underestimates the survival, however the Kaplan-

meier estimate is based on very few observations.


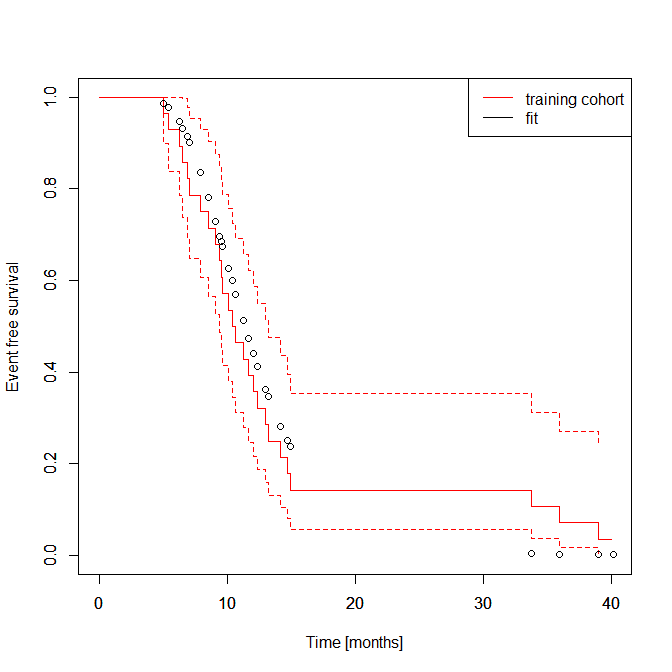


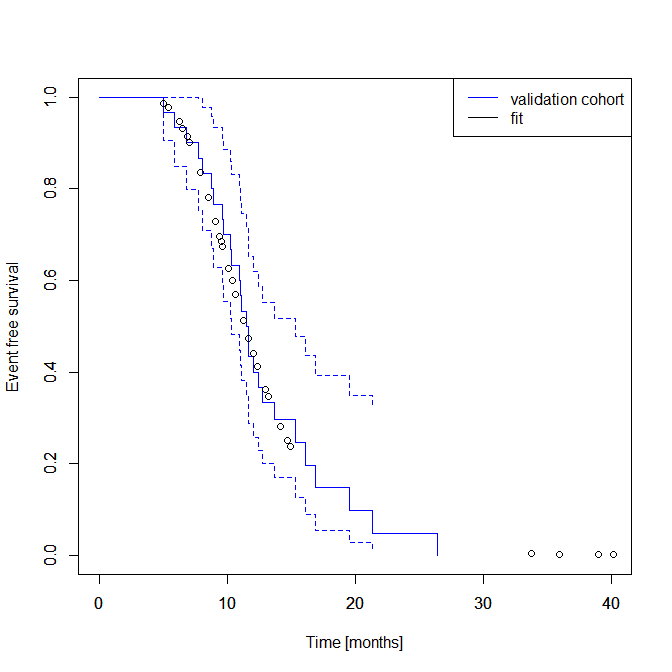

Supplement: Supplementary file 3 — Additional file 3. Details on the multivariate Cox regression model for FDG PET radiomics. [file 13550_2020_669_MOESM3_ESM.docx]
